# Uterus transplantation: Experimental animal models and recent experience in humans

**DOI:** 10.4274/tjod.66809

**Published:** 2015-03-15

**Authors:** Sadık Şahin, Selçuk Selçuk, Mustafa Eroğlu, Ateş Karateke

**Affiliations:** 1 Zeynep Kamil Training and Research Hospital, Clinic of Gynecologic and Pediatric İstanbul, Turkey

**Keywords:** Uterus transplantation, experimental studies, ischemia-reperfusion, immuno-suppressive drugs

## Abstract

Uterus transplantation has been considered as an alternative management modality in the last few years for adoption or gestational surrogacy for women with absence of uterus due to congenital or acquired reasons. Surrogacy is legal in only a few countries because of ethical, social and legal issues. Up to date, a total of 11 uterus transplantation cases have been reported in which uteri were harvested from ten live donors and one donor with brain death. After unsuccessful attempt of first uterus transplantation, many studies have been conducted in animals and these experimental models enabled our knowledge to increase on this topic. First experimental studies were performed in rodents; later uterus transplantation was accomplished in sheep, pigs and rabbits. Recently, researches in non-human primates have led the experience regarding transplantation technique and success to improve. In this review, we reviewed the experimental animal researches in the area of uterus transplantation and recent experience in humans.

## INTRODUCTION

In recent years, uterus transplantation (UTx) in women without uterus due to congenital or acquired reasons have been experimentally performed in eleven women until today. Ideal candidates for UTx include women with congenital absence of the uterus (Mayer-Rokitansky-Küster-Hauser syndrome) and those who have undergone hysterectomy (because of benign or malignant causes and postpartum hemorrhage). First UTx from a living donor has been performed by Fageeh et al. in 2000, but they had to remove the uterus 3 months after the transplantation because of prolapsus and necrosis^(1)^. However, this first transplantation in human has been performed without sufficient animal studies. After the first failed UTx, several studies have been conducted on animal models and our knowledge on this topic began to increase. Later, the first UTx from a donor with brain death has been carried out in our country by Ozkan et al. in 2011^(2)^. This transplantation was technically successful, although it was not resulted in a live birth. It was reported in a recent series of nine cases on this subject that at a 6-month clinical follow-up, viability of uterus continued in seven women who undergone transplantation and these women began to menstruate regularly^(3)^. Ischemia-reperfusion injury is one of the most important factors affecting success of organ transplantation. The other factors which play a role in the success include duration of exposure of the organ to be transplanted to warm and cold ischemia, realization and continuity of flow in the vessels that were anastomosed to the recipient and efficiency of immunosuppressive drugs^(4)^.

## ISCHEMIC DAMAGE AND EXPERIMENTAL MODELS IN UTERUS TRANSPLANTATION

**Figure 1:**
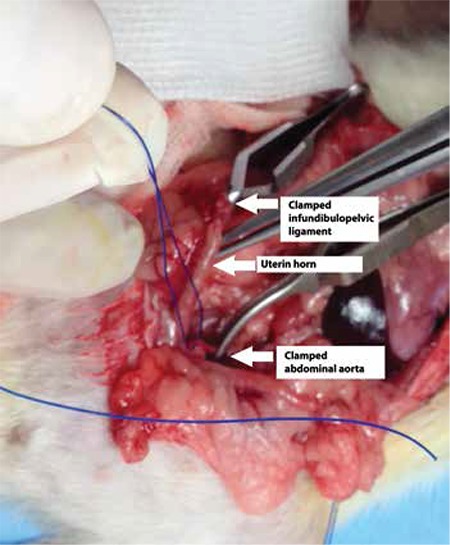
Ischemia- reperfusion model of rat uteri

Ischemia-reperfusion injury causes severe injuries in the transplanted organs. Ischemic damage which occurring during organ transplantation can be categorized in three parts: 1. Warm ischemia time I (WI-1): period between clamping of the vessels to be removed from the donor and cold perfusion, 2. Cold ischemia (CI) time; period until anastomosis of the organ which is cooled with a cold perfusion solution to the recipient, and 3. Warm ischemia time II (WI-II); ischemic period beginning with the perfusion of the organ with vessels anastomosed^(5)^. In UTx from donors with brain death, WI-I period is expected to be longer than in those from living donors. Because, in case of donors with brain death, vital organs (liver, kidney etc) are removed first from the donors and transplanted^(6)^. In addition, if UTx is planned from these donors, the uterus is the last organ to be transplanted since it is non-vital and this prolonges the duration of the exposure to warm ischemia. Therefore, experimental studies on the prevention of ischemic damage to the uterus in transplantation performed from donors with brain death are crucial. We demonstrated in one study that tacrolimus which is an immunouppressive drug shows antiinflammatory and antioxidating effects, decreasing ischemia-reperfusion injury to which the uterus is exposed^(7)^. In this model, we carried out abdominal aorta dissection in 2-3-month-old Wistar rats. We inserted one bulldog (Aesculap) clamp at the distal abdominal aorta to provide uterus ischemia and two bulldog clamps under both ovaries to prevent collateral blood flow (Figure 1). We provided reperfusion after 30 minutes by removing the clamps. We divided the rats into two groups. We administered tacrolimus to one of the groups before ischemia and to the other group before reperfusion. Uteri of the rats were removed after reperfusion and activities of malondealdehid, superoxide dismutase, glutathione and catalase, which are the markers of oxidative damage, were studied. Furthermore, histopathological tissue examination was performed. We observed in both groups that tacrolimus decreased ischemia-reperfusion damage to the uterus biochemically and histologically. However, this protective effect was more significant in the group which received tacrolimus before ischemia than in the group which received it before reperfusion. We believe that this model would be important in terms of allowing trial of new drugs and antioxidants in prevention of warm ischemia-reperfusion injury. In a study conducted on rats investigating the duration of uteral exposure to warm ischemia, 240-minute warm ischemia was reported to reduce the success of transplant^(8)^. Nevertheless, the maximum duration of warm ischemia which the uterus can expose is still unclear.

Another important issue in the transplantation from persons with brain death is cold ischemic injury. In the transplantation from these donors the organ exposes to CI until it is transported to the recepient. In the UTx performed in Turkey, uterus was the first organ removed from the donor and CI time was defined as 30 minutes^(2)^. However, CI period is expected to be longer in UTx from donors with brain death. We are currently working on a model investigating the effects of the solutions of Custodiol and University of Wisconsin on uterus and their mechanisms in various ways. Studies demonstrated that human myometrium is cold ischemia-resistant for 12 hours^(9)^. However, prolongation of WI-I time due to lack of blood circulation in the body of the donors with cardiac death and subsequent effects on the uterus during CI are issues that should be investigated.

## UTERUS TRANSPLANTATION IN EXPERIMENTAL ANIMALS

UTx has been performed in several species of experimental animals. Studies that first began with mice and rats have been expanded and successfully applied on animals such as sheep, pigs and finally primates close to human (baboon, macaque)^(10)^. We can classify these studies as autologous, syngeneic and allogeneic according to the transplantation performed among different strains of the same species.

## TYPES OF TRANSPLANTATION

Autologous transplantation is transplantation of the uterus removed from an animal and transplated again to the same animal. Syngeneic transplantation is transplantation of the uterus removed from an animal to the animal of same species (e.g. transplantation of a uterus from a Wistar rat to another Wistar rat). These two models are important especially in terms of showing the effects of ischemia-reperfusion injury which may be encountered in transplantation surgery.

Syngeneic UTx can be categorized into two types as heterotopic and orthotopic UTx. Heterotopic UTx is performed through end-to-side anastomosis of the uterus and vessels of the donor to the aorta and vena cava vessels of the recipient. In this model, which has been tested on mice, cervical part of the transplanted uterus has been left free in the abdomen or anastomosed to the anterior abdominal wall^(11,12)^. Pregnancy and birth have been reported in the trans-myometrial embryo transfer following the transplantation in both of the models. In addition, viable pregnancy after transplantation has been reported in rats with anastomosis to the skin in heterotopic transplantation model^(13)^. In the rats undergone orthotopic UTx, only cornual part of the uterus of the recipient was left, hysterectomy was performed and, fundal and vaginal part of the transplated uterus was anastomosed to vaginal part and the cornual region of the recipient which has been left intact. So far, syngeneic UTx has been performed only in mice and rats. Pregnancy resulted in live birth following normal copulation has been demonstrated in the rats undergone orthotopic UTx^(14)^.

Autologous transplantation has been tested in sheep, pig, baboon and macaque models. Live birth following spontaneous copulation has been reported in a sheep undergone uterus autotransplantation in which ovary and tuba have been unilaterally protected^(15)^.

Allogeneic transplantation is a transplantation model among different strains of the same species (i.e. transplantation of the uterus from Wistar rats to Lewis rats). Rejection of transplanted organ can help us determine efficiency of immunosuppressive drugs and obtain data in terms of pregnancy outcomes for the UTx operations which can be performed in human. In this model, which has been tested first in mice, immunosuppressive drugs have not been given and histopathological changes in the transplanted uterus have been evaluated. Respective dominance of macrophages, neutrophils, and cytotoxic T cells has been observed in the transplantaed uteri^(16)^. Later, cyclosporine which is an immunosuppressive agent has been tested in mice undergone allogeneic transplantation, but this could not prevent rejection of the organ^(17^). In another study, rejection of organ has been shown to be prevented by the use of tacrolimus in the allogeneic transplantation performed in rats^(18)^. In our study, we demonstrated that tacrolimus prevented tissue damage by showing antiinflammatory and antioxidating effects^(7)^. In another study, pregnancy after copulation has been shown for the first time in rats which were given tacrolimus following UTx. This was important since it was the first pregnancy reported after allogeneic UTx^(19)^. Afterwards, cyclosporine has been used in sheep after UTx and pregnancy has been reported following embryo transfer^(20)^.

## UTERUS TRANSPLANTATION IN NON-HUMAN PRIMATES

Autologous and allogeneic UTx have been tested in non-human primates such as baboon, rhesus macaque and cynomolgus macaque. First, orthotopic UTx has been performed in Saudi Arabia with end-to-end anastomosis of the uterine vessels, but most of these attempts have resulted in vascular thrombosis. Then, end-to-side anastomosis of the internal iliac and uterine vessels have been performed and a success rate of 90% has been reported^(1)^. Again, it has been found in another UTx trial with baboons that shortening of the operation time and providing organ perfusion under ideal conditions increase postoperative survival and number of subjects with menses. However, pregnancy following copulation has not been observed in these baboons due to severity of intraabdominal adhesions^(21)^. In another study with baboons undergone allogeneic UTx, survival rates of the donors and recipients were 100% in the first 5 days of the operation, although tacrolimus alone could not prevent rejection of organs. In addition, no menses were observed in these animals undergone UTx^(22)^. Recently, pregnancy and live birth have been reported in a macaque which underwent autotransplantation with unilaterally protected ovary and tuba^(23)^. However, although the combination of tacrolimus, mycophenolate and corticosteroids have been found to prevent rejection of the organ in a cynomolgus macaque undergone autotransplantation, so far no live birth occurred in non-human primates undergone autotransplantation^(24)^.

## UTERUS TRANSPLANTATION IN HUMANS

The first UTx from a living donor has failed due to necrosis developed following vascular thrombosis^(1)^. The first UTx from cadaver has been performed in our country^(2)^. Besides, advantages of the organs revomed from cadavers include having wider and longer vascular pedicles. These organs also have disadvantages due to greater exposure to ischemic damage and systemic inflammatory response after brain death^(6)^. Tests should be carried out before UTx in order to exclude papillomavirus infection, uteral and endometrial pathologies and to demonstrate HLA similarity. Transplantations from cadavers bring risks because limited time will not always allow these tests to be performed^(5)^. In addition, kidney transplantations from a living donor have been proven to be more long lasting than those from cadavers^(25)^. However, since UTx is a non-vital transplantation, hysterectomy should be planned during occurence of living birth. Furthermore, due to the fact that living donors are usually mothers of the recipients, age of the donors should be expected as older than 50 years and risk of endarteritis is likely to be more^(26)^. Therefore, the question of whether pregnancy and live birth from a living donor following UTx will have disadvantage compared to the transplantation from cadavers is yet to be answered. In a UTx case by Ozkan et al., donor was a 22-year-old woman with brain death and human leucocyte antigen (HLA) typing, human immune deficiency virus (HIV), typing hepatitis B and C serology, transabdominal ultrasonography, cervical smear screening and rapid HPV typing of the donor have been carried out^(2)^. There was no condition to hinder removal of the donor’s uterus on these tests performed before transplantation. Hypogastric arteries and veins of the donor have been end-to-side anastomosed to the external iliac artery and veins of the recipient. Uterosacral ligaments of the donors have been fixed to the presacral peritoneum of the recipient and also round ligament of the donor has been connected to the inguinal ligament of the recipient. Rejection of the organ has not been reported within 12 months with triple immunosuppressant (tacrolimus, mycophenolate mofetil and prednisolone). Pregnancy has been observed following transfer of frozen embryo, but this has ended with fetal loss in an early week of gestation^(27)^. A Swedish team performed UTx from two mothers to their daughters in November 2012^(28)^. In these transplantations, uteri of the donors have been removed with ovarian veins and vascular pedicles until the bufircation region. End-to-side anastomosis has been applied to the external iliac artery and veins of the recipient. Ovarian vein has been unilaterally anastomosed to the uterine vein or external iliac vein of the recipient in order to provide a better drainage. The donors and recipients have been preoperatively given antibiotic prophylaxis with a single dose of piperacillin/tazobactam (4 g) and this therapy has been continued postoperatively as three times a day for one day in the donor and the same dose for three days in the recipient. Prophylaxis of thrombosis has been achieved with dalteparin (5000 IU) for 21 days in the donor and 42 days in the recipient. In addition, acetylsalicylic acid (75 mg) has been administered to the recipients for 6 months. Combination of tacrolimus, mycophenolate mofetil and prednisolone has been used as immunosuppressive regimen in the recipients^(3)^. Afterwards, 6-month data have been reported following nine UTx cases from the living donors (mostly mothers of the recipients). It has been reported that viability of the transplanted uteri continued in seven of these nine patients at the end of six months and these women began to menstruate regularly. Mild rejection episodes have been reported in four of these seven recipients, but these episodes have been successfully overcome with corticosteroids therapy of 7-10 days. Transplanted uterus in two women were rejected. One of these has resulted from acute bilateral thrombotic uterine artery occlusion and another one from treatment-resistant intrauterine infection. Only in one donor uretero-vaginal fistula has occurred and this has been corrected with a second operation about 4 months after transplantation surgery. Brannstrom et al. demonstrated that UTx from a living donor is a reliable method despite prolongation of the surgery time^(3)^.

Today, gestational surrogacy and adoption are available options in women with infertility of uterus origin. These options do not include the surgical risks that may be brought by transplantation to the donors and recipients. However, number of the countries legally allowing surrogacy is limited because of social and religious reasons. UTx may be considered as an option after its success is proven in these countries. UTx is distinguished from other transplantations, because it is a non-vital method. Furthermore, UTx is a method requiring long-term immunosuppressive therapy in the post-transplantation period and during the time from possible pregnancy until birth and this may bring additional risks both for the expectant mother and fetus. Preeclampsia, growth retardation and increased risk for prematurity have been reported in pregnancies of the women undergone kidney transplantation^(29)^. Therefore, ethical debates on UTx are crucial. UTx candidates and their partners should be informed in detail about the alternative methods, the risks that the surgery may bring and those may be encountered during a possible pregnancy. So far, 8 of 11 UTx operations have been reported to be successful. It has been demonstrated in 14.000 infants who were born from the women given immunosuppressive drugs during pregnancy that these drugs did not increase the rate of the congenital anomalies(^30)^.

## CONCLUSION

Experimental animal models and a few clinical experiences have provided data suggesting that Utx is a reliable method. Ethical aspects and risks of this transplantation which is not vital should be told to the candidates in details. UTx is still in the trial stage and actual success of the transplantations from living donors and cadavers will be achieved with possible pregnancies which will result in live births.
